# Detecting inbreeding depression in structured populations

**DOI:** 10.1073/pnas.2315780121

**Published:** 2024-04-30

**Authors:** Eléonore Lavanchy, Bruce S. Weir, Jérôme Goudet

**Affiliations:** ^a^Department of Ecology and Evolution, University of Lausanne, Lausanne 1015, Switzerland; ^b^Population Genetics and Genomics group, Swiss Institute of Bioinformatics, University of Lausanne, Lausanne CH-1015, Switzerland; ^c^Department of Biostatistics, University of Washington, Seattle WA 98195

**Keywords:** inbreeding, inbreeding depression, population structure

## Abstract

Measuring inbreeding and its consequences on fitness is central for many areas in biology including human genetics and the conservation of endangered species. However, there is no consensus on the best method, neither for quantification of inbreeding itself nor for the model to estimate its effect on specific traits. We simulated traits based on simulated genomes from a large pedigree and empirical whole-genome sequences of human data from populations with various sizes and structures (from the 1,000 Genomes project). We compare the ability of various inbreeding coefficients (F) to quantify the strength of inbreeding depression: allele-sharing, two versions of the correlation of uniting gametes which differ in the weight they attribute to each locus and two identical-by-descent segments-based estimators. We also compare two models: the standard linear model and a linear mixed model (LMM) including a genetic relatedness matrix (GRM) as random effect to account for the nonindependence of observations. We find LMMs give better results in scenarios with population or family structure. Within the LMM, we compare three different GRMs and show that in homogeneous populations, there is little difference among the different F and GRM for inbreeding depression quantification. However, as soon as a strong population or family structure is present, the strength of inbreeding depression can be most efficiently estimated only if i) the phenotypes are regressed on F based on a weighted version of the correlation of uniting gametes, giving more weight to common alleles and ii) with the GRM obtained from an allele-sharing relatedness estimator.

## Significance

Inbreeding depression is the reduction of individuals’ fitness caused by inbreeding and is traditionally quantified via (generalized) linear regressions of the phenotype on the inbreeding coefficient. While this approach might be adequate for homogeneous populations, it could lead to a biased estimation of the strength of inbreeding depression in structured populations. In this manuscript, we compare the classical linear model approach to a mixed model accounting for population structure by including genomic relationship matrices. We address two additional questions: i) Which inbreeding coefficient is most suitable for estimating inbreeding depression? ii) Which relatedness matrix allows for the best correction for structure? We compare eight different inbreeding coefficients and three different relatedness matrices in populations of various sizes and structures.

Inbreeding is the result of mating between relatives and is often associated with reduced fitness, a phenomenon called inbreeding depression (ID) and which was observed in many different species such as humans (1, 2), other animals (3–6), and plants (7).

Many different methods have been developed for inbreeding quantification and there is no consensus on which one is the best (8–14). The classical approach was first proposed by Sewall Wright in 1922 and makes use of pedigrees (called hereafter $F_{\mathrm{PED}}$) (15). With the advances in sequencing technologies, genomic-based inbreeding coefficients (hereafter called $F_{\mathrm{genomic}}$) have been developed. Among these, some coefficients rely on the comparison between observed and expected heterozygosity such as $F_{\mathrm{HOM}}$ (16, 17), the expected allele-sharing between individuals such as $F_{\mathrm{AS}}$ (13), or on the correlation between uniting gametes such as $F_{\mathrm{UNI}}$ (18). In addition to estimating the realized inbreeding coefficient and requiring no prior knowledge of the pedigree of the population, these genomic estimates are simple and straightforward to compute and do not require whole-genome sequencing (WGS) data; a few thousand SNPs are usually sufficient for reliable inbreeding estimation in humans (10). However, they also have a disadvantage: They usually require quadratic moments of allelic proportions (except for $F_{\mathrm{AS}}$). These moments have expectations that are complex functions of allele probabilities and coancestry coefficients, leading to biased estimates (13). Another inbreeding coefficient was proposed by McQuillan et al. (19): $F_{\mathrm{ROH}}$ uses runs of homozygosity (ROHs), long homozygous stretches as a proxy for identical-by-descent (IBD) segments within individuals (19). A model-based approach relying on hidden Markov models has also been developed for detecting IBD segments (20) by identifying homozygous-by-descent (HBD) segments. This model is the basis for many other model-based IBD segment detection methods such as BCFTools (21), BEAGLE (22), and RZooRoH (23). The inbreeding coefficient estimated with these model-based approaches will be called $F_{\mathrm{HBD}}$ from now on. One advantage of these methods is that they can be used when very few individuals are sampled, as the reference is the genome of the individual rather than the variation in the population at each variable site. However, it has been shown that these coefficients, and especially $F_{\mathrm{ROH}}$, are sensitive to SNP density and the parameters used to search for ROHs or HBD segments. There is no consensus on what is the most suitable set of parameters at present (24, 25).

How to quantify ID, although central to conservation genetics for decades (14, 26) (more details and references in *SI Appendix* for this paper), is still debated. This debate includes two subquestions: Which statistical model should be employed? And which F? Regarding the model, the classical approach consisted of the use of linear regression of the phenotypes on the inbreeding coefficient (LM). However, other models have been utilized, such as generalized LMs (GLMs) with various link functions. In 2019, Nietlisbach et al. (11) compared different models and found that the common GLM with logit link did not allow for accurate ID strength estimation. They propose using GLM with logarithm link functions. Ultimately, the type of model is largely dependent on the distribution of the trait.

Regarding the choice of which F is more accurate for quantifying ID, many studies have demonstrated that $F_{\mathrm{genomic}}$ yields better results than $F_{\mathrm{PED}}$ (27–30). However, some studies found $F_{\mathrm{UNI}}$ to be more accurate than $F_{\mathrm{ROH}}$ (12), while others found that $F_{\mathrm{ROH}}$ provided the best estimates of ID (11, 27, 29, 31). In 2020, Caballero et al. (9) used simulations and included several populations with different histories: They found that the optimal F actually depends on how large is the population. $F_{\mathrm{ROH}}$ did a better job at quantifying ID in populations with small effective size while $F_{\mathrm{UNI}}$ was better at predicting ID estimates in populations with large effective sizes. This result was later confirmed by Alemu et al. (8) who used SNP-array empirical cattle data for several groups of allelic frequencies and concluded that $F_{\mathrm{UNI}}$ and $F_{\mathrm{GRM}}$ ($F_{I}$ and $F_{\mathrm{III}}$, respectively in ref. 18) are better at quantifying homozygosity at rare alleles while $F_{\mathrm{ROH}}$ and $F_{\mathrm{HOM}}$ are better for alleles at intermediate frequencies and correlate better with whole-genome homozygosity. Indeed, recessive deleterious alleles, which are thought to be responsible for ID, should segregate at low frequencies in large populations as a result of purifying selection. On the contrary, in small populations, drift can increase the frequency of deleterious recessive alleles to intermediate frequencies, making $F_{\mathrm{ROH}}$ and $F_{\mathrm{HOM}}$ more suitable for detecting ID. Indeed, in the simulations conducted by Yengo et al. (12), rare alleles always caused negative effects on fitness (referred to as DEMA, for Directional Effect of Minor Alleles). The authors showed that $F_{\mathrm{HOM}}$ (and thus $F_{\mathrm{AS}}$ since they have similar properties) is sensitive to DEMA while $F_{\mathrm{UNI}}$ and $F_{\mathrm{ROH}}$ are not. They also showed via simulations that all estimates of ID are somewhat sensitive to population structure, $F_{\mathrm{UNI}}$ being the least affected. They recommend estimating ID using linkage disequilibrium (LD) score and minor allele frequency (MAF) bins, and summing the ID estimates from these bins as an overall estimate of ID for the trait.

In this paper, we simulated traits based on both simulated and empirical WGS human data from populations with varying sizes and structures. We show that some *F* are more sensitive to population structure and DEMA than others. We confirm only some of Yengo et al. (12) results. Importantly, we show that accounting for the nonindependence of observations with a mixed model via an allele-sharing based genomic relationship matrix (GRM) (rather than the standard GCTA GRM) and using a modified version of $F_{\mathrm{UNI}}$ which gives more weight to common alleles resolves most of the issues raised by Yengo et al. (12).

## Results

All the figures presented in the main text picture the scenario where allele additive effect sizes and dominance coefficients are proportional to MAF and where there is a directional additive effect of minor alleles (DEMA) (i.e., the ADD & DOM & DEMA scenario from *SI Appendix*, Table S1 and Fig. S1). The results for the other scenarios are shown and discussed in *SI Appendix*, Figs. S10–S17 and Tables S3–S6).

### Simulated Pedigrees.

**Fig. 1.**
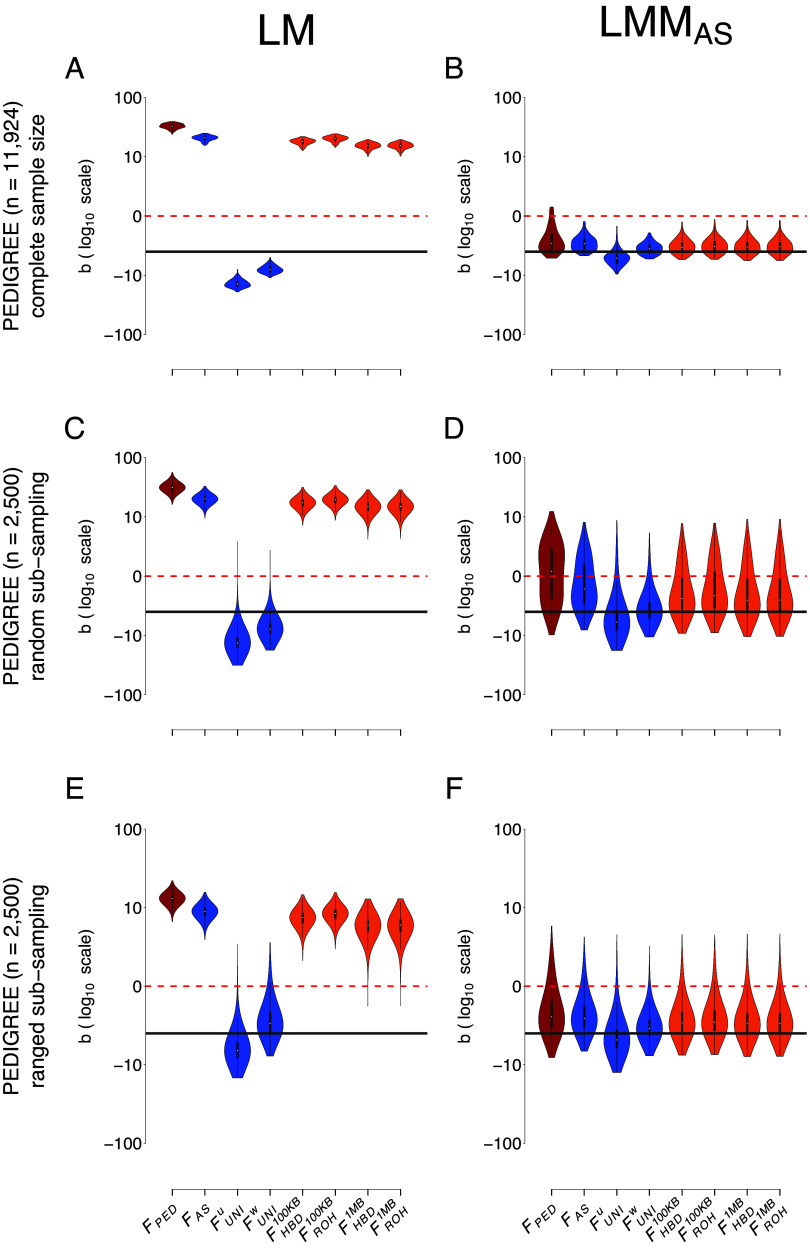
Comparison of the estimation of ID strength (b) among different F estimates and two models in the PEDIGREE population. Each column represents a regression model. The first column depicts the simple linear regression (LM), and the second column depicts the LMM with the allele sharing relatedness matrix as a random component (LMM_AS_). The first row represents the complete simulated population (11,924 individuals, *A* and *B*). The second row shows the random subsampling (2,500 individuals, *C* and *D*). The third row shows the ranged subsampling (2,500 individuals, *E* and *F*). Inbreeding estimates presented in this graph are $F_{\mathrm{PED}}$, $F_{\mathrm{AS}}$, $F_{\mathrm{UNI}}^{U}$, $F_{\mathrm{UNI}}^{W}$, $F_{{\mathrm{HBD}}_{100\mathrm{KB}}}$, $F_{{\mathrm{ROH}}_{100\mathrm{KB}}}$, $F_{{\mathrm{HBD}}_{1\mathrm{MB}}}$, and finally $F_{{\mathrm{ROH}}_{1\mathrm{MB}}}$. For *A* and *B*, violin plots show the distribution of the ID strength estimates (b) among the 100 simulation replicates. For *C*–*F*, violin plots represent the distribution of the ID strength estimates (b) for the 10,000 simulation and subsampling replicates (100 subsampling replicates for each of the 100 simulation replicates). The solid dark gray line is the true strength of ID (b=−3). The dashed red line represents the absence of ID (b=0), meaning that we failed to detect ID in any replicate above this line. Note that all panels are in $log_{10}$ scale and that all replicates converged.

Fig. 1 presents the ID strength estimates (*b*, see *Materials and Methods*) for the different inbreeding coefficients (*F*), with two regression models in the PEDIGREE populations. The first column shows *b* estimated with the simple LM and the second column shows *b* estimated with LMM including the allele-sharing GRM as a random factor (LMM_AS_). The first row shows results for the complete PEDIGREE population (n=11,924). The second row shows results for a reduced sample size of the PEDIGREE population (n=2,500, meant to match the size of the 1KG WORLD population) where subsampled individuals were chosen completely randomly. The third row also shows results for a reduced sample size of the PEDIGREE population (n=2,500) but these individuals were selected to represent the entire spectrum of inbreeding values. The violin plots show *b* estimates distributions among the simulation replicates (100 replicates for the complete population, 10,000 replicates for both subsampled populations). The solid dark gray line is the true strength of ID (*b* = −3). The dashed red line represents the absence of ID (*b*=0), indicating that ID was not detected in any replicate above this line. RMSE values associated with both models and populations are shown in Table 1. Strikingly, in the PEDIGREE population, all F resulted in a biased estimation of b with the simple LM, whatever the sample size (Fig. 1*A*, *C*, and *E* and Table 1). The inclusion of a GRM as a random factor allowed for the correction of nonindependence of observations and greatly improved b estimation (Fig. 1*B*, *D*, and *F* and Table 1). In the complete PEDIGREE population, we see little difference between the three GRMs we tested (Fig. 1*B* vs. *SI Appendix*, Fig. S6 *A* and *B* and Table 1): all F yielded efficient (we use efficient to describe an estimate with low RMSE, thus which is unbiased and has low variance) estimates of b when used inside a LMM, except for $F_{\mathrm{UNI}}^{U}$ that slightly overestimates the strength of ID while $F_{\mathrm{PED}}$ slightly underestimates it. This suggests that large sample sizes (here 11,924 individuals) combined with a mixed model allow efficient ID estimation regardless of the *F* used. The three mixed models, however, perform less efficiently when the sample size is reduced, as we demonstrate with both subsampled PEDIGREE populations (n=2,500): many replicates produced estimates above zero for b (Fig. 1 *D* and *F* and *SI Appendix* Fig. S6 *C*–*F* and Table 1). RMSEs were particularly large for $F_{\mathrm{PED}}$, $F_{{\mathrm{HBD}}_{100\mathrm{KB}}}$, and $F_{{\mathrm{ROH}}_{100\mathrm{KB}}}$ with the mixed model using the unweighted GCTA GRM (LMM_*GCTA*^*U*^_) (*SI Appendix*, Fig. S6*D* and Table 1). Additionally, increasing the variance of subsampled individuals’ F (i.e., ranged subsampling) led to better estimates of b with reduced variance among replicates compared to random subsampling (Fig. 1*D* vs. *F*: *SI Appendix*, Fig. S6 *C* vs. *E* and *D* vs. *F* and Table 1). To assess the performance of the different models with even smaller sample sizes, often seen in wild and nonmodel species, we simulated pedigrees with only 50, 100, 250, and 500 individuals (*SI Appendix*, Fig. S7). With all sample sizes, the simple LM produces biased estimates (*SI Appendix*, Fig. S7 *A*, *E*, *I*, and *M*). Including a GRM improved the estimation of b, but less so than for larger pedigree sizes (*SI Appendix*, Fig. S7 *B*–*D*, *F*–*H*, *J*–*L*, and *N*–*P*). The lowest RMSE was obtained with LMM_AS_, but the difference with both GCTA-based GRMs was marginal.

**Table 1.** RMSE on b estimate in the PEDIGREE population

Model	Population	$F_{\mathrm{PED}}$	$F_{\mathrm{AS}}$	$F_{\mathrm{UNI}}^{U}$	$F_{\mathrm{UNI}}^{W}$	$F_{{\mathrm{HBD}}_{100\mathrm{KB}}}$	$F_{{\mathrm{ROH}}_{100\mathrm{KB}}}$	$F_{{\mathrm{HBD}}_{1\mathrm{MB}}}$	$F_{{\mathrm{ROH}}_{1\mathrm{MB}}}$
LM	PEDIGREE (complete)	34.82	22.71	10.17	4.17	19.93	22.22	17.4	17.44
LMM_AS_	PEDIGREE (complete)	1.62	1.27	1.89	0.87	1.07	1.12	1.11	1.11
LMM_*GCTA*^*W*^_	PEDIGREE (complete)	1.62	1.27	1.89	0.87	1.07	1.12	1.11	1.11
LMM_*GCTA*^*U*^_	PEDIGREE (complete)	1.58	1.28	1.85	0.88	1.08	1.12	1.08	1.08
LM	PEDIGREE (random sub)	33.84	22.20	10.41	4.47	19.53	21.72	17.24	17.28
LMM_AS_	PEDIGREE (random sub)	4.01	2.97	3.82	1.83	2.57	2.73	2.56	2.57
LMM_*GCTA*^*W*^_	PEDIGREE (random sub)	4.01	2.97	3.82	1.83	2.57	2.73	2.56	2.57
LMM_*GCTA*^*U*^_	PEDIGREE (random sub)	>1,000	2.75	3.44	1.78	>1,000	>1,000	>1,000	>1,000
LM	PEDIGREE (ranged sub)	15.22	11.04	3.46	1.61	9.58	10.52	8.13	8.15
LMM_AS_	PEDIGREE (ranged sub)	2.09	1.82	2.13	1.26	1.61	1.67	1.58	1.58
LMM_*GCTA*^*W*^_	PEDIGREE (ranged sub)	2.09	1.82	2.13	1.26	1.61	1.67	1.58	1.58
LMM_*GCTA*^*U*^_	PEDIGREE (ranged sub)	>1,000	1.69	2.05	1.24	>1,000	>1,000	1.53	1.54

These values are for the complete ADD & DOM & DEMA scenario. See *SI Appendix*, Tables S3–S6 for the other scenarios.

### 1,000 Genomes Project.

**Fig. 2.**
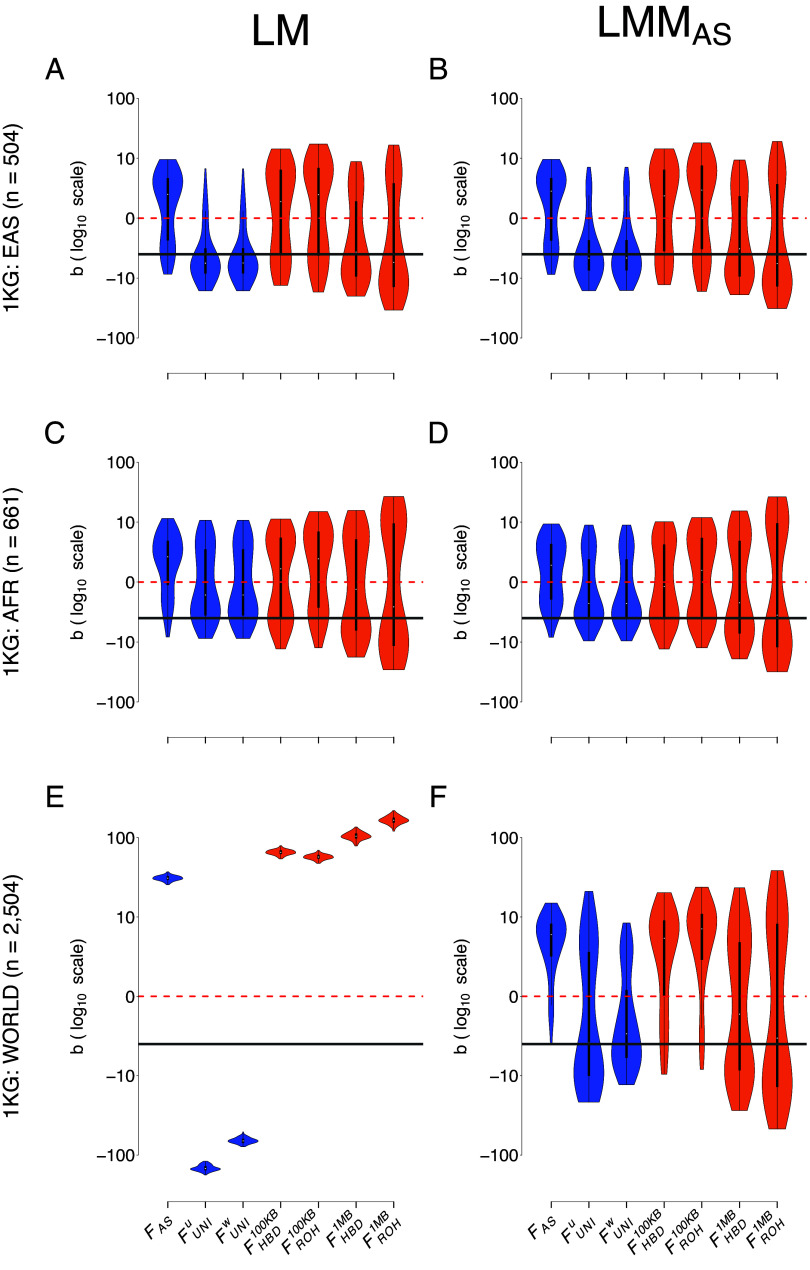
Comparison of the estimation of ID strength (b) among different F estimates and two models in the three populations from the 1,000 Genomes project. Each column represents a regression model. The first column depicts the simple linear regression (LM) and the second column depicts the LMM with the allele-sharing relatedness matrix as a random component (LMM_AS_). The three rows correspond to the three populations from the 1,000 Genomes project: EAS on *A* and *B*, AFR on *C* and *D*, and WORLD on *E* and *F*. Inbreeding estimates presented in this graph are $F_{\mathrm{AS}}$, $F_{\mathrm{UNI}}^{U}$, $F_{\mathrm{UNI}}^{W}$, $F_{{\mathrm{HBD}}_{100\mathrm{KB}}}$, $F_{{\mathrm{ROH}}_{100\mathrm{KB}}}$, $F_{{\mathrm{HBD}}_{1\mathrm{MB}}}$, and finally $F_{{\mathrm{ROH}}_{1\mathrm{MB}}}$. Violin plots show the distribution of the ID strength estimates (b) among the simulation 100 replicates. The solid dark gray line is the true strength of ID (b=−3). The dashed red line represents the absence of ID (b=0), meaning that we failed to detect ID in any replicate above this line. Note that all panels are in $log_{10}$ scale and that all replicates converged.

Fig. 2 illustrates the estimates of ID strength (*b*) for the different inbreeding coefficients (*F*), when using either a LM or a LMM for two subsets of the 1,000 Genomes Project: East-Asian ancestry (EAS) and African ancestry (AFR), as well as for the entire world population (WORLD). It has the same structure as Fig. 1. RMSE values associated with both models and populations can be found in Table 2. Interestingly, we see little difference between LM and LMM and the different GRMs when there is no structure among the samples even with small sample sizes (EAS: Fig. 2 *A* and *B* vs. *SI Appendix*, Fig. S8 *A* and *B* and Table 2; AFR: Fig. 2 *C* and *D* vs. *SI Appendix*, Fig. S8 *C* and *D* and Table 2). Similarly to what was observed for the PEDIGREE population, when some structure exists (population structure in the WORLD population compared to family structure in the PEDIGREE population), the simple LM fails to accurately estimate the strength of ID, regardless of the F (Fig. 2*E* and Table 2). In contrast to the pedigree population showing no difference between the three GRMs (Fig. 1 and *SI Appendix*, Fig. S6), the most efficient estimates of b are obtained only with the LMM_AS_ model and with $F_{\mathrm{UNI}}^{W}$ in the highly structured WORLD population (Fig. 2*F* vs. *SI Appendix*, Fig. S8 *E* and *F* and Table 2). In fact, the models including the $GCTA^{W}$ and $GCTA^{U}$ matrices cannot efficiently estimate *b* with any of the inbreeding coefficients: even though *b* with $F_{\mathrm{UNI}}^{W}$ are unbiased, the variance is very large (Fig. 2*F* and *SI Appendix*, Fig. S8 and Table 2). In addition, several replicates did not converge when both $GCTA^{W}$ and $GCTA^{U}$ models were used which was never the case with the $GRM_{\mathrm{AS}}$. Numbers of such replicates are indicated in the figures’ legend and in *SI Appendix*, Tables S7–S9. Similarly to what was done for the PEDIGREE population, we subsampled individuals from the WORLD population to test the different models with smaller sample sizes (50, 100, 250, and 500, as shown in *SI Appendix*, Fig. S9). The results are very similar to those observed in the large WORLD population. Unsurprisingly, the simple LM fails to adequately quantify ID with all sample sizes (*SI Appendix*, Fig. S9 *A*, *E*, *I*, and *M*), and the most efficient estimation of b is obtained using LMM_AS_ and $F_{\mathrm{UNI}}^{W}$ (*SI Appendix*, Fig. S9 *C*, *G*, *K*, and *O*). Here, mixed models using either LMM_*GCTA*^*W*^_ or LMM_*GCTA*^*U*^_ fail to accurately quantify b with any F (*SI Appendix*, Fig. S9 *C*, *D*, *G*, *H*, *K*, *L*, *O*, and *P*).

**Table 2.** RMSE on b estimate in the three 1,000 Genomes Project populations: EAS, AFR, and WORLD

Model	Population	$F_{\mathrm{AS}}$	$F_{\mathrm{UNI}}^{U}$	$F_{\mathrm{UNI}}^{W}$	$F_{{\mathrm{HBD}}_{100\mathrm{KB}}}$	$F_{{\mathrm{ROH}}_{100\mathrm{KB}}}$	$F_{{\mathrm{HBD}}_{1\mathrm{MB}}}$	$F_{{\mathrm{ROH}}_{1\mathrm{MB}}}$
LM	EAS	5.55	4.9	4.86	7.14	7.93	6.19	10.58
LMM_AS_	EAS	5.67	4.68	4.64	7.41	8.22	6.12	10.39
LMM_*GCTA*^*W*^_	EAS	5.67	4.68	4.64	7.28	8.06	6.11	10.39
LMM_*GCTA*^*U*^_	EAS	5.48	4.74	4.71	7.1	7.87	6.18	10.57
LM	AFR	5.93	4.81	4.81	6.03	7.21	7.21	13.12
LMM_AS_	AFR	5.15	4.07	4.07	5.46	6.2	7.15	13.1
LMM_*GCTA*^*W*^_	AFR	5.15	4.07	4.07	>1,000	>1,000	7.16	13.1
LMM_*GCTA*^*U*^_	AFR	5.78	4.42	4.42	5.92	6.93	7.2	13.11
LM	WORLD	32.91	142.95	62.21	67.42	59.15	107.67	169.73
LMM_AS_	WORLD	8.63	8.34	4.17	9.15	10.97	8.78	14.6
LMM_*GCTA*^*W*^_	WORLD	9.84	>1,000	>1,000	11.19	13.92	>1,000	>1,000
LMM_*GCTA*^*U*^_	WORLD	18.18	>1,000	>1,000	27.52	26.91	>1,000	>1,000

These values are for the complete ADD & DOM & DEMA scenario. See *SI Appendix*, Tables S3–S6 for other scenarios.

### Comparing Inbreeding Coefficients.

With both the LM and LMM_AS_ models in the three populations from the 1,000 Genomes Project (EAS, AFR, and WORLD, Fig. 2*A*–*F*) and for the LM in the PEDIGREE population, $F_{\mathrm{AS}}$ is consistently underestimating the strength of ID, particularly when there is strong structure (WORLD: Fig. 2 *E* and *F*). It is because DEMA is included in the model and strongly influences the quantification of ID by $F_{\mathrm{AS}}$. In the absence of a DEMA, $F_{\mathrm{AS}}$ produces efficient estimates (*SI Appendix*, Figs. S10–S13). In addition, $F_{\mathrm{AS}}$ is sensitive to the dominance effects being proportional to MAF but to a lesser extent and in the opposite direction (*SI Appendix*, Fig. S10 vs. S11). Concerning the other SNP-based *F*, $F_{\mathrm{UNI}}^{U}$ is constantly overestimating the strength of ID and is the most sensitive to population structure: its variance is much larger compared to $F_{\mathrm{UNI}}^{W}$ in the structured WORLD population and with all models (Fig. 2*F* and Table 2). Interestingly, the variance of $F_{\mathrm{UNI}}^{U}$ is affected only when allele effect sizes and/or dominance coefficients are proportional to MAF, but not by DEMA (*SI Appendix*, Figs. S10–S17). In contrast, $F_{\mathrm{UNI}}^{W}$ is the least sensitive to allele effect sizes or dominance coefficients proportional to MAF and DEMA (*SI Appendix*, Figs. S10–S17), which makes it the most appropriate *F* for estimating ID (Fig. 2*F* and Table 2). Since the difference between $F_{\mathrm{UNI}}^{W}$ and $F_{\mathrm{UNI}}^{U}$ is the weight given to rare and common alleles, we conducted the same analyses (including the re-estimation of both F and GRMs estimation) on the WORLD population but excluding loci with MAF < 0.05 and showed that there is no difference between $F_{\mathrm{UNI}}^{W}$ and $F_{\mathrm{UNI}}^{U}$ when rare alleles are removed (*SI Appendix*, Fig. S18). Concerning the *F* calculated from ROHs and HBD segments, there is not much difference between PLINK and BCFTools except for the variance among *b* estimates, which is slightly smaller with BCFTools compared to PLINK (Fig. 2*A*–*F* and Table 2). In addition, focusing on recent inbreeding by including only large segments (here larger than 1MB) yielded better results in the WORLD population (Fig. 2*F*). Since BCFTools is a model-based HBD approach, there is no mandatory length requirement. In light of this, we also estimated $F_{\mathrm{HBD}}$ based on HBD segments without any size restrictions, and the results are similar to those obtained using $F_{{\mathrm{HBD}}_{100\mathrm{KB}}}$ (*SI Appendix*, Fig. S19). We also quantified ID with ROHs and HBD segments larger than 5MB but it did not improve the estimation of b (*SI Appendix*, Fig. S19).

### Comparing Genetic Relatedness Matrices.

**Fig. 3.**
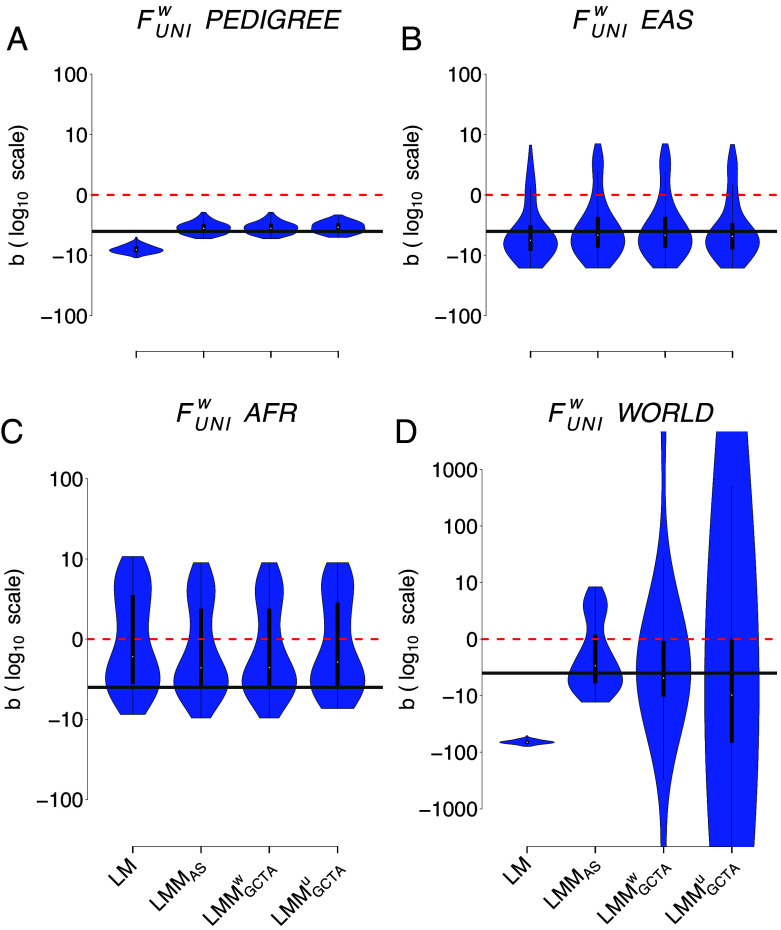
Comparison of the ID strength estimates (b) with $F_{\mathrm{UNI}}^{W}$ in the four populations with four different models. The four models are i) the simple linear regression (LM), ii) the LMM with the allele-sharing relatedness matrix as a random factor, iii) the LMM with the weighted GCTA relatedness matrix as a random factor, and iv) the LMM with the unweighted GCTA relatedness matrix as a random factor. Panel *A* shows the simulated PEDIGREE population, panel *B* the EAS population, panel *C* the AFR population and finally panel *D* the WORLD population. Note that all panels are in $log_{10}$ scale. Also note that LMM did not converge for some replicates (yielding estimated b values above 1,000 or below −1,000). Percentages of replicates which did not converge: panel *D* (WORLD): 21% for $GRM_{{\mathrm{GCTA}}^{W}}$; 20% for $GRM_{{\mathrm{GCTA}}^{U}}$.

Since we identified $F_{\mathrm{UNI}}^{W}$ as the best inbreeding coefficient to quantify ID, Fig. 3 contrasts the four different models for this coefficient in the four populations: each panel corresponds to one population. As mentioned above, there is almost no difference among the different GRMs in the extremely large complete PEDIGREE population (Fig. 3*A* and Table 1) and between any of the models in the two homogeneous populations (EAS and AFR) (Fig. 3 *B* and *C* and Table 2). However, in the highly structured WORLD population, LMM_AS_ gives the most efficient result due to its smaller variance and RMSE (Fig. 3*D* and Table 2).

### Distribution of Additive and Dominance Effects.

We found a difference between the three LMMs only because the scenario presented in the main text includes effect sizes and dominance coefficients proportional to causal markers’ MAF as well as DEMA. When none of these three parameters are included, there is little difference between the three LMMs (*SI Appendix*, Fig. S10 *B*, *F*, *J*, and *N* vs. *C*, *G*, *K*, and *O* vs. *D*, *H*, *L*, and *P* and Tables S3–S6). Additional simulations were conducted without additive and dominance coefficients proportional to loci’s MAF and DEMA to assess their impact on ID detection. These other scenarios are explored and discussed in details in *SI Appendix*, Figs. S10–S17.

Finally, we also investigated i) the effect of the LDMS stratification method proposed by Yengo et al. (12) (*SI Appendix*, Figs. S10–S17) but found that it improves results only with the simple LM and not as much as the LMM_AS_ model and ii) the effect of using intermediate frequencies causal loci (*SI Appendix*, Fig. S20) which reduced the variance in b estimates for all inbreeding coefficients.

### Application to an Empirical Dataset.

As an illustration of our methods, we analyze adult mass and bill depth of a metapopulation of house sparrows in northern Norway using a dataset from Niskanen et al. (32) (analyses for other morphological traits are given in *SI Appendix*). For mass (Table 3), the slope associated with $F_{\mathrm{UNI}}^{W}$ is b=−2.39,P=0.02 in the simple LM. The model the authors of the paper used (32) is a LMM with the island and year nested in islands as random effects and results in b=−1.98,P=0.05. Using only $GRM_{\mathrm{AS}}$ as a random effect makes the slope steeper and more significant: b=−2.86,P=0.007. If we include the $GRM_{\mathrm{AS}}$, the island, and year nested in island (the full model), the results are very similar to using $GRM_{\mathrm{AS}}$ only: b=−2.85,P=0.006. For bill depth, the slope associated with $F_{\mathrm{UNI}}^{W}$ is positive (Table 3) and significant for the LM (b=0.27,P=0.039), which suggests the presence of outbreeding depression for this trait. With the LMM_AS_, however, the slope is shallower and not significant (b=0.22,P=0.106). Including islands and years (nested in islands) as random effects shows a similar pattern, and the full model makes the slope for $F_{\mathrm{UNI}}^{W}$ shallower and its *P*-value larger.

**Table 3.** Analysis of adult mass and bill depth from 1,786 adult sparrows

Mass	Int.	Sex	$F_{\mathrm{UNI}}^{W}$	$V_{\;\text{I}}$	$V_{\;\text{Y:I}}$	$V_{A}$	$V_{E}$	$P_{F}$
LM	33.0	−1.39	−2.39				4.59	0.02
LMM_AS_	34.3	−1.41	−2.86			1.56	3.02	0.007
LMM	32.9	−1.38	−1.98	0.15	0.27		4.27	0.050
$\;{\text{LMM}}_{\;\text{Full}}$	34.3	−1.40	−2.85	0.01	0.17	1.45	2.92	0.006
Bill depth	Int.	Sex	F	$V_{\;\text{I}}$	$V_{\;\text{Y:I}}$	$V_{A}$	$V_{E}$	$P_{F}$
LM	8.1	0.04	0.27				0.08	0.039
LMM_AS_	8.1	0.03	0.22			0.04	0.04	0.106
LMM	8.1	0.04	0.24	0.00	0.01		0.07	0.068
$\;{\text{LMM}}_{\;\text{Full}}$	8.1	0.03	0.23	0.00	0.01	0.04	0.04	0.084

LM: simple linear model with sex and $F_{\mathrm{UNI}}^{W}$ as explanatory variables. LMM_AS_: LMM with sex and $F_{\mathrm{UNI}}^{W}$ as fixed effects and $GRM_{\mathrm{AS}}$ as random effect. LMM: linear mixed model with sex and $F_{\mathrm{UNI}}^{W}$ as fixed effect and island and year nested in island as random effects. $\;{\text{LMM}}_{\;\text{Full}}$: LMM with sex and $F_{\mathrm{UNI}}^{W}$ as fixed effects and island, year nested in island and $GRM_{\mathrm{AS}}$ as random effects. $V_{\;\text{I}}$: variance component of island effect; $V_{\;\text{Y:I}}$: variance component for year nested in island; $V_{A}$: additive variance; $V_{E}$: residual variance; $P_{F}$: *P*-value for the slope b of $F_{\mathrm{UNI}}^{W}$ to be 0.

## Discussion

By analyzing the phenotypes of a large simulated pedigreed polygamous population with strong family structure as well as subsets of the 1,000 genomes project (33), we demonstrated that, despite population or family structure, ID strength can be efficiently estimated if the data are analyzed with a mixed model including the genomic relationships among individuals as a random effect. While the use of a relationship matrix as a random factor in mixed models for quantitative genetics analyses is standard (34), and GRMs have been used for the estimation of heritability (18, 35–37) and in GWAS (18, 37–41) for a long time, it is seldom used to quantify ID [see McQuillan et al. (42) for a notable exception; we did not discover any follow-up papers using a similar approach until Nishio et al. (43) who used the GCTA based GRM in 2023, although Stoffel et al. (44) use a model with breeding values as random effects]. We evaluate the ability of the LMM approach (including different GRMs) to quantify ID and compare it to the classical LM. First, we show that for most scenarios, ID is better estimated with LMM than with a simple LM and second, compared to other GRMs in LMM, the allele sharing–based GRM provides the most efficient results, especially for small sample sizes and samples with a high family or population structure. In addition, among the several inbreeding estimators tested, $F_{\mathrm{UNI}}^{W}$ proved to be the most reliable coefficient to quantify ID. We further confirm these results with an empirical dataset and show that using the LMM_AS_ and $F_{\mathrm{UNI}}^{W}$ can significantly alter the results of ID quantification compared to using a simple LM.

We observed trivial differences among the different models when there is no population structure (i.e., in the EAS and AFR populations). However, as soon as there is some structure (the WORLD and PEDIGREE populations) the classical LM completely fails to estimate b regardless of the inbreeding coefficient used. This result is concordant with Yengo et al. (2017) (12) where the authors quantified ID using a simple LM and demonstrated that $F_{\mathrm{HOM}}$ (whose properties are very similar to $F_{\mathrm{AS}}$), $F_{\mathrm{UNI}}^{U}$ and two different $F_{\mathrm{ROH}}$ were sensitive to population structure. As for the comparison of three LMMs, they perform equally when the population structure is weak (familial structure in the PEDIGREE population and weak population structure in EAS and AFR) or when there are very large sample sizes (11,924 individuals from the complete PEDIGREE population). Although samples of this size are common for research on humans, they will seldom be found in wild populations. We therefore subsampled the PEDIGREE population to 2,500 individuals in order to investigate the effect of a smaller sample size and the range of inbreeding of the samples. We used two types of subsampling: i) random subsampling where individuals were chosen completely randomly and ii) ranged subsampling where individuals were chosen to maximize the range of F in the sampled population. As expected, when we subsampled individuals from the PEDIGREE population, RMSE values associated with b estimation increased slightly for both LMM_AS_ and LMM_*GCTA*^*W*^_ mixed models and we failed to detect ID in some replicates. Accordingly, despite improving the estimation of b relative to the LM, the LMM_AS_ model lacks power with smaller sample sizes (50, 100, 250, 500, and 2,500 individuals): it failed to detect ID by estimating b≥0 in 26% of replicates and several thousands of individuals would be required to detect ID efficiently (i.e., in all replicates) as Keller et al. (26) and Caballero et al. (45) previously pointed out. With the LMM_*GCTA*^*U*^_ mixed model, all inbreeding coefficients but $F_{\mathrm{AS}}$ and $F_{\mathrm{UNI}}$ had convergence issues, suggesting that the LMM_*GCTA*^*U*^_ mixed model is the least robust of the three mixed models. As expected, randomly subsampling individuals leads to a larger variance of b estimates compared to the ranged subsampling scheme, indicating that maximizing the variance of samples’ F improves the estimation of b, although it is not obvious how such sampling could be done in nonmonitored natural populations.

When we add a strong population structure in addition to small sample size (2,504, 500, 250, 100, and 50 individuals) from the highly structured WORLD population), we observe striking differences between the three different GRMs. The LMM including the allele-sharing–based GRM (LMM_AS_) resulted in the most efficient estimations of b. In addition, the mixed models with both $GRM_{{\mathrm{GCTA}}^{U}}$ and $GRM_{{\mathrm{GCTA}}^{W}}$ did not converge for a high percentage of replicates (compared to 0% for LMM_AS_) emphasizing that LMM_AS_ is the best model for quantifying ID in highly structured populations and that it can also be applied to small sample sizes. This is because the allele-sharing–based GRM is a better estimator of kinship compared to both GCTA matrices (10, 46). Indeed, what the $GRM_{\mathrm{AS}}$ estimates is the actual kinship in the population, based on how many alleles individuals share. In contrast, what both $GRM_{{\mathrm{GCTA}}^{W}}$ and $GRM_{{\mathrm{GCTA}}^{U}}$ estimate is a combination of individual kinship, their mean kinship with the other individuals, and the overall mean kinship in the population [see Equation **3** in Goudet et al. (46)]. Consequently, since the kinship itself is better estimated with $GRM_{\mathrm{AS}}$, the nonindependence of observations (and thus the population structure) is better accounted for with LMM_AS_ which leads to better b estimates. Importantly, the inclusion of a GRM in the ID estimation model is not limited to simple LMs. Even though we used only LMs in this study, any type of GLM can incorporate a GRM as a random factor. Consequently, this method can be applied to any trait distribution. Furthermore, by including the GRM-based random factor, the nonindependence of observations is better accounted for than by including the population as a random factor, and no prior knowledge of the population structure is required.

### Comparing *F*.

Concerning the different inbreeding coefficients, we found $F_{\mathrm{UNI}}^{W}$ to be the best F for quantifying ID. Indeed, $F_{\mathrm{UNI}}^{W}$ was the only coefficient we tested which was not sensitive to either additive and dominance effect sizes being proportional to MAF or DEMA resulting in the least biased estimation of b. On the contrary, we found that $F_{\mathrm{UNI}}^{U}$ was influenced by the dominance effect sizes being proportional to MAF and by population structure. In $F_{\mathrm{UNI}}^{U}$ estimation, the rare alleles associated with large dominance effect sizes add noise in the estimation of b. Similarly, when there is population structure, rare alleles which have a strong influence on $F_{\mathrm{UNI}}^{U}$ are likely to be private alleles which will strongly bias population-specific allelic frequencies and eventually $F_{\mathrm{UNI}}^{U}$ estimation. Importantly, $F_{\mathrm{UNI}}^{U}$ performed as well as $F_{\mathrm{UNI}}^{W}$ when we filtered on MAF>0.05 for F and all GRMs estimation. This is because $F_{\mathrm{UNI}}^{U}$ uses the average of ratios, which results in loci with small MAF strongly influencing the outcome. When these rare loci are filtered out, the estimated F is no longer biased. This explains why Yengo et al. (12) found that $F_{\mathrm{UNI}}^{U}$ was the best F for quantifying ID with a homogeneous subset of the UK biobank dataset: They filtered on MAF>0.05 leading to $F_{\mathrm{UNI}}^{U}$ estimation not being influenced by rare alleles with strong additive and/or dominance effect sizes. Concerning $F_{\mathrm{AS}}$, we found that it was very sensitive to DEMA. This result is also concordant with Yengo et al. (12) who found that $F_{\mathrm{HOM}}$ (with properties very similar to $F_{\mathrm{AS}}$) was sensitive to DEMA. In this paper, the authors explain that this sensitivity is due to $F_{\mathrm{HOM}}$ (and thus $F_{\mathrm{AS}}$) correlating strongly with minor allelic count which will create a spurious association with ID in the presence of DEMA. However, $F_{\mathrm{AS}}$ resulted in the most efficient estimates of b when DEMA was not included in the model, suggesting that it is the best F to estimate inbreeding for neutral regions, as was argued by Zhang et al. (13). Finally, we found that ROHs and HBD segments based F, namely $F_{\mathrm{ROH}}$ and $F_{\mathrm{HBD}}$, performed poorly: underestimating the strength of ID (positive b) or displaying very large variance among replicates. This result is in contradiction with Kardos et al. (29, 47) and Nietlisbach et al. (11) who found that $F_{\mathrm{ROH}}$ and $F_{\mathrm{HBD}}$ were better at quantifying ID compared to SNPs-independent based F. However, Alemu et al. (8) and Caballero et al. (9) showed the best F actually depends on the history of the population. Indeed, they showed that $F_{\mathrm{ROH}}$ and $F_{\mathrm{HBD}}$ and to a lesser extent $F_{\mathrm{HOM}}$ were better at quantifying homozygosity at loci with common alleles. On the contrary, $F_{\mathrm{UNI}}^{U}$ was better at quantifying homozygosity at rare alleles. Alemu et al. (8) and Caballero et al. (9) propose that in populations with low effective sizes, selection is weaker and deleterious alleles may be able to reach intermediate frequencies as a result of drift. Therefore both $F_{\mathrm{ROH}}$ and $F_{\mathrm{HBD}}$ (and $F_{\mathrm{HOM}}$ in their analyses) should perform better in such populations. In our study, the standard scenario (with no ADD, no DOM, and no DEMA) mimics what happens in such small populations and we found that $F_{\mathrm{ROH}}$, $F_{\mathrm{HBD}}$, and $F_{\mathrm{AS}}$ (which has similar properties to $F_{\mathrm{HOM}}$) performed better than $F_{\mathrm{UNI}}^{U}$ (which is the $F_{\mathrm{UNI}}$ they tested) in the highly structured WORLD population and to a lesser extent in the family structured PEDIGREE population. With homogeneous populations, we do not observe any difference between these inbreeding coefficients. Nevertheless, this is consistent with Alemu (8) results, as they used families which consequently create structure. On the contrary, in populations with a large effective size, selection maintains deleterious alleles at low frequencies which explains why Yengo et al. (12) found that $F_{\mathrm{UNI}}$ was the best F with the large UK biobank dataset and this is consistent with what we have found with the ADD & DOM & DEMA scenario which mimics what happens in populations with large effective sizes.

## Conclusion

We showed that the more efficient method for estimating ID is to use a mixed model with an allele-sharing-based relatedness matrix as a random component but $F_{\mathrm{UNI}}^{W}$ as the inbreeding coefficient to predict ID. The most commonly used GRM ($GRM_{{\mathrm{GCTA}}^{U}}$) results in biased and highly variable estimates of b in structured populations. We stress that even if the results are greatly improved by using the allele-sharing GRM and $F_{\mathrm{UNI}}^{W}$, the variance among replicates is still large and we failed to detect ID in several replicates ($\hat{b}$≥0) in the highly structured WORLD population (for all sample sizes) as well as in the small and slightly admixed AFR population. Therefore, detecting ID of the magnitude commonly found and that we simulated requires very large sample sizes with several thousand individuals, particularly in structured populations. Unfortunately, this might be hardly feasible for wild and/or endangered populations.

## Materials and Methods

All scripts used in this manuscript can be found on GitHub.

### Simulated Pedigrees.

We simulated a polygamous pedigree from a dioecious population with overlapping generations (hereafter called PEDIGREE) using custom R scripts. The population started from 500 founders (equal numbers of males and females) and followed a polygamous mating system: Female fertilities per time interval were drawn from a Poisson distribution with parameter λ=1, mortality rate per time interval was set to 0.5, and only 10% of the males were allowed to reproduce at each time step. Matings were recorded for 25 time steps, resulting in a pedigree of 11,924 individuals (over 25 time steps).

In order to simulate the genotypes of the individuals, we proceeded in two steps. We used the mspms wrapper to the msprime software (48) to simulate the two haplotypes containing L=650,000 loci for each founder individual. The L loci were uniformly distributed along a constant recombination map 20M long. For each reproduction event, the number of cross-overs was first drawn from a Poisson distribution and then randomly positioned along the genome. The nonfounder genotypes were then obtained by drawing two gametes: one from each parent. For each gamete, the allele at the first locus is selected at random between the two alleles of the parent. The alleles at the next loci along the chromosome are copied from the chromosome with the chosen allele at the first locus until a recombination event occurs, at which point the alleles are copied from the other chromosome until the next crossing-over or the end of the chromosome.

In order to investigate the effect of using more realistic smaller sample sizes, we subsampled 2,500 individuals from the PEDIGREE population. We performed two types of subsampling: i) a random subsampling where individuals were subsampled completely randomly, ii) a stratified subsampling where we sought to retain the widest range of inbreeding coefficients in the subsampled population. Consequently, for this stratified subsampling individuals with $F_{\mathrm{UNI}}^{W}$≥0.2 were always included and individuals with $F_{\mathrm{UNI}}^{W}$<0.2 were randomly selected until the population reached the desired size. 100 replicates were performed for each subsampling. To test the methods with even smaller sample sizes, we simulated smaller pedigree (resulting in 50, 100, 250, and 500 individuals) with lower numbers of founders (8, 16, 40, and 80, respectively).

### 1000 Genomes.

In order to extend our conclusions to smaller sample sizes and populations with stronger structure (which are common in wild and/or endangered species), we used empirical data from phase 3 from the 1,000 Genomes project (33). We considered i) a small sample from a homogeneous population with a small effective size represented by 504 individuals from the superpopulation with East-Asian ancestry (EAS), ii) a small sample from a population with some admixture and larger effective population sizes represented by 661 individuals from the superpopulation with African-ancestry and admixed individuals (AFR) and finally iii) a larger sample from a population with larger effective size and with genetic structure (global $F_{\mathrm{ST}}=0.083$) comprising all the 2,504 individuals (hereafter called WORLD) and represented by five superpopulations: individuals with EAS, AFR, European ancestry (EUR), admixed American ancestry (AMR), and finally South-Asian ancestry (SAS). A more detailed description of the samples can be found at the 1,000 Genomes Project website. To extend our findings to even smaller sample sizes, we subsampled the WORLD populations to 50, 100, 250, and 500 individuals. In each subsampling, we ensured that the entire range of F was covered and that similar numbers of individuals were subsampled from each continent.

### Simulated Traits.

We simulated traits based on Eq. **1** following ref. 12: we consider a trait y whose phenotype is partly determined by the genotypes at Lc causal loci with $h^{2}=0.8$. We assume these loci to be biallelic, with one allele encoding for an increase in the trait value (the plus allele) and the other encoding for a decrease in trait value (the minus allele). Dominance was also considered since ID occurs only if there is directional dominance: when heterozygotes at loci encoding for the trait are closer on average to the homozygote for the plus allele (34). If gene effects are purely additive or if dominance is not directional, there is no ID. Finally, we assume no epistasis between loci and no genotype-environment interaction.

For individual j, $y_{j}$ is the individual trait value (its phenotype), calculated as the sum of allelic and genotypic effects over causal loci, an environmental effect and μ, the average trait value among all individuals. At locus l, $x_{\mathrm{jl}}$ is the minor allele count (MAC) ∈{0,1,2} of individual j. $a_{l}$ represents the additive effect size of the alternate allele at locus l. $d_{l}$ is the dominance effect size, the deviation of the heterozygous genotype from the mean of the two homozygotes. Finally, $\epsilon_{j}$ is the environmental contribution to the phenotype of individual j, drawn from a normal distribution.

[1] $$\begin{array}{c} y_{j}=\mu +\sum_{l=1}^{\mathrm{Lc}}x_{\mathrm{jl}}a_{l}+\sum_{l}^{\mathrm{Lc}}x_{\mathrm{jl}}\left(2-x_{\mathrm{jl}}\right)d_{l}+\epsilon_{j}. \end{array}$$

The strength of ID b was set to −3 in all simulations, as in Yengo et al. (12). The value corresponds to an average reduction in trait value of 0.75 SD for an offspring resulting from a mating between full-siblings.

We used Eq. **1** to simulate traits with varying architectures. To avoid causal markers with extremely low frequencies, we first excluded loci with MAF≤0.01 for both the EAS and AFR populations and loci with MAF≤0.001 for both the PEDIGREE and WORLD populations. We then simulated traits using 1,000 randomly chosen SNPs (after MAF filtering). We initially drew both the raw (i.e., unscaled) additive effect sizes of the alternate allele and the raw dominance effect sizes from a uniform [0,1] distribution (other distributions were explored with almost no effect on the results). As we expect alleles causing ID to be counterselected and thus removed or maintained at a low frequency (proportionally to their detrimental effect), the raw effect sizes were scaled inversely to MAF $a_{j}=\;{\text{raw}}_{\mathrm{aj}}/p_{j}$ to mimic purifying selection. We also scaled the dominance effects inversely to the locus expected heterozygosity $d_{j}=\;{\text{raw}}_{\mathrm{dj}}/\left(2p_{j}\left(1-pj\right)\right)$. In addition, we attributed the same sign to the effect sizes of all minor alleles in order to include what Yengo et al. (12) called DEMA (12). However, in order to investigate the effect of the parameters mentioned above, we also simulated traits where the additive and dominance effect sizes were left unchanged $a_{j}=\;{\text{raw}}_{\mathrm{aj}}$ and $d_{j}=\;{\text{raw}}_{\mathrm{dj}}$ and without DEMA. A summary of all the simulated scenarios can be found in *SI Appendix*, Table S1. In addition, graphical representation of the additive effect sizes and dominance coefficients distribution under these different scenarios can be found in *SI Appendix*, Fig. S1.

### Individual Inbreeding Coefficients.

We estimated individual inbreeding coefficients using several methods whose properties were recently described in detail in Zhang et al. (13). Regarding the figures and tables presented in the main text, we do not filter on MAF for any of the F estimates. We use one allele-sharing-based estimator of inbreeding, hereafter called $F_{\mathrm{AS}}$ and described in refs. 13 and 46:

[2] $$\begin{array}{c} F_{AS_{j}}=\frac{\sum_{l=1}^{L}\left(A_{\mathrm{jl}}-A_{\mathrm{Sl}}\right)}{\sum_{l=1}^{L}\left(1-A_{\mathrm{Sl}}\right)}, \end{array}$$

where $A_{\mathrm{jl}}$ indicates the identity of the two alleles an individual j carries at locus l: one for homozygous and 0 for heterozygous and $A_{\mathrm{Sl}}$ is the average allele-sharing proportion at locus l for pairs of individuals j,k,j≠k.

Then, we compare two versions of $F_{\mathrm{UNI}}$ (initially described in ref. 18) and which measure the correlation between uniting gametes. The first version (hereafter called $F_{\mathrm{UNI}}^{U}$) is the original $F_{\mathrm{UNI}}$ (18) measured as the average of ratios over SNPs (which attributes equal weight (1/L) to all loci and results in loci with rare alleles having larger influence on the estimated F):

[3] $$\begin{array}{c} F_{{\mathrm{UNI}}_{j}}^{u}=\frac{1}{L}\sum_{l=1}^{L}\frac{x_{\mathrm{jl}}^{2}-\left(1+2p_{l}\right)x_{\mathrm{jl}}+2p_{l}^{2}}{2p_{l}\left(1-p_{l}\right)}. \end{array}$$

Similarly to Eq. **1**, $x_{\mathrm{jl}}$∈{0,1,2} is the MAC of individual j at locus l and $p_{l}$ is the derived allele frequency at locus l.

The second version (hereafter called $F_{\mathrm{UNI}}^{W}$) is a modified version of $F_{\mathrm{UNI}}$ which measures the ratio of averages (rather than the average of ratios) and thus gives more weight to loci with larger expected heterozygosity (i.e., with MAF close to 0.5). We are not aware of other investigations using the ratio of averages estimator $F_{\mathrm{UNI}}^{W}$ in the context of ID estimation.

[4] $$\begin{array}{c} F_{{\mathrm{UNI}}_{j}}^{w}=\frac{\sum_{l=1}^{L}\left(x_{\mathrm{jl}}^{2}-\left(1+2p_{l}\right)x_{\mathrm{jl}}+2p_{l}^{2}\right)}{\sum_{l=1}^{L}2p_{l}\left(1-p_{l}\right)}. \end{array}$$

We also used four identical-by-descent (IBD) segments based *F*. We identified runs of homozygosity (ROHs) with PLINK (17) and default parameters. We also modeled homozygous-by-descent (HBD) segments with BCFTools (21). For both methods, we selected ROHs or HBD segments based on their size: either larger than 100Kb: $F_{{\mathrm{ROH}}_{100\mathrm{KB}}}$ and $F_{{\mathrm{HBD}}_{100\mathrm{KB}}}$ or larger than 1Mb: $F_{{\mathrm{ROH}}_{1\mathrm{MB}}}$ and $F_{{\mathrm{HBD}}_{1\mathrm{MB}}}$. For both methods, the inbreeding coefficients were simply estimated as the fraction of genome falling within ROHs or HBD segments.

Finally, in the PEDIGREE population, we used the pedigree-based inbreeding coefficient: $F_{\mathrm{PED}}$ (15).

All inbreeding coefficients were estimated separately for each population of the 1,000 Genomes Project (EAS, AFR, WORLD) and using only the polymorphic SNPs in each population and population-specific allelic frequencies (for both $F_{\mathrm{UNI}}$). Consequently, the same individual might have different $F_{\mathrm{genomic}}$ in the EAS and the WORLD population. This influenced only trivially the IBD segments-based inbreeding coefficients ($F_{\mathrm{ROH}}$ and $F_{\mathrm{HBD}}$) but influenced greatly $F_{\mathrm{AS}}$ (though the rank of inbreeding among individuals was perfectly conserved) and both $F_{\mathrm{UNI}}$ (for which the rank of inbreeding among individuals was not conserved). Comparison among the different inbreeding coefficients per population can be found in *SI Appendix*, Figs. S2–S5). More details can be found in ref. 13.

### Estimation of ID: b.

We estimated the strength of ID (hereafter defined as b) using two different models. In the first model, b was estimated as the slope of regression of phenotypes on the different inbreeding coefficients with a classical LM:

[5] $$\begin{array}{c} {\hat{b}}_{\mathrm{LM}}=Cov\left(Y,F\right)/Var\left(F\right), \end{array}$$

where Y is the vector of trait values and F is the vector of individual inbreeding coefficient estimates.

In the second model, we estimate b as the fixed effect coefficient associated with the inbreeding coefficient in the following LMM:

[6] $$\begin{array}{c} Y=bX+\omega +\epsilon , \end{array}$$

where Y is the vector of trait values, X is a matrix with two columns, the first containing ones and the second the individual inbreeding coefficients, ω is the random component of the mixed model with ω∼N(0,τK), K being the GRM and τ the additive variance component. Finally, ϵ is the individual residual variance and is defined as $\epsilon ∼\sigma^{2}I_{n}$. From this, b is estimated as follows:

[7] $$\begin{array}{c} {\hat{b}}_{\mathrm{LMM}}={\left(X^{\prime }V^{-1}X\right)}^{-1}X^{\prime }V^{-1}Y \end{array}$$

with $V=\tau K+\sigma^{2}I_{n}$ (49). We compared three GRMs we estimated using all loci (no MAF filtering). The first mixed model included a GRM derived from allele sharing (10), hereafter called LMM_AS_. We used the R Hierfstat (50) package to estimate K and the R gaston package (51) to estimate V and b. We could not use GCTA software to run the mixed model for this GRM because its leading eigenvalue is negative which the Choleski decomposition algorithm used for matrix inversion in GCTA cannot handle (it requires a positive definite matrix), while the Schur decomposition algorithm used in gaston can. We note that the GCTA GRM is not positive definite (one eigenvalue is 0), but the matrix to invert in the mixed model is not the GRM itself but $V=\tau K+\sigma^{2}I_{n}$ which becomes positive definite and can be inverted if the heritability is smaller than one.

The second mixed model used the GCTA weighted GRM (10, 52). Similarly to $F_{\mathrm{UNI}}^{W}$, this matrix uses the ratio of averages. For this model, we used GCTA and the R SNPrelate package to estimate the GRM. We then used the R gaston package for estimating V and b with the LMM.

Finally, the third mixed model used the GCTA unweighted GRM (18) which (similarly to $F_{\mathrm{UNI}}^{U}$) utilizes the average of ratios and thus gives equal weight to all loci. For this model, we used GCTA to estimate the GRM. We then estimated V and b with the LMM implemented in the R gaston package.

Note that the average information-restricted maximum likelihood (AIREML) fitting method we used in the LMM is an iterative procedure and should result in unbiased estimates. In some cases, the model did not converge and gave highly biased b. For each scenario, regression model, and population, the number of replicates which did not converge can be found in *SI Appendix*, Tables S7–S9.

### Application to an Empirical Dataset.

A metapopulation of house sparrows (*Passer domesticus*) from several islands in Northern Norway has been monitored since 1993 and Niskanen et al. (32) investigated ID on several traits and made available phenotype and genotype data on more than 3,100 adult individuals. The dataset is ideal to illustrate our method as individuals belong to many islands and the data contain slight genetic structure and some individuals are highly related (see *SI Appendix* for further details).

We used only morphological phenotypes, as they can be analyzed with LMs. We removed information from nonautosomes (scaffold 32) but otherwise kept all SNPs to avoid biases when filtering for minor allele frequencies and LD (46). We filtered out individuals who were not present as adults in one of the eight studied islands, as was done in the original analysis (32). The dataset used for analysis contained 1,786 individuals genotyped at 181,529 SNPs. We compared the results of a simple LM with Sex and $F_{\mathrm{UNI}}^{W}$ as explanatory variables, to the LMM_AS_ model with Sex and $F_{\mathrm{UNI}}^{W}$ as fixed effects. We also present two additional LMMs: one with island and year nested in island as random effects, as done in the original article, and a “full” mixed model with all the random effects mentioned above. Estimates for the LMs were obtained with the lm function of R, while estimates for the mixed models were obtained with the lmer function of the lme4 package or the lmm.aireml function of the gaston package if the model contained a GRM. To test whether b, the slope associated with $F_{\mathrm{UNI}}^{W}$, was significantly different from 0, we used the score.fixed.linear function of the gaston package.

## Supplementary Material

Appendix 01 (PDF)

## Data Availability

Previously published data were used for this work (33).
